# Sequencing and expression of two arsenic resistance operons with different functions in the highly arsenic-resistant strain *Ochrobactrum tritici *SCII24^T^

**DOI:** 10.1186/1471-2180-8-95

**Published:** 2008-06-13

**Authors:** Rita Branco, Ana-Paula Chung, Paula V Morais

**Affiliations:** 1 IMAR-Laboratory of Microbiology 3004-517 Coimbra, Portugal; 2 Department of Biochemistry, University of Coimbra, 3001-401 Coimbra, Portugal

## Abstract

**Background:**

Arsenic (As) is a natural metalloid, widely used in anthropogenic activities, that can exist in different oxidation states. Throughout the world, there are several environments contaminated with high amounts of arsenic where many organisms can survive. The most stable arsenical species are arsenate and arsenite that can be subject to chemically and microbiologically oxidation, reduction and methylation reactions. Organisms surviving in arsenic contaminated environments can have a diversity of mechanisms to resist to the harmful effects of arsenical compounds.

**Results:**

The highly metal resistant *Ochrobactrum tritici *SCII24 was able to grow in media with arsenite (50 mM), arsenate (up to 200 mM) and antimonite (10 mM). This strain contains two arsenic and antimony resistance operons (*ars*1 and *ars*2), which were cloned and sequenced. Sequence analysis indicated that *ars*1 operon contains five genes encoding the following proteins: ArsR, ArsD, ArsA, CBS-domain-containing protein and ArsB. The *ars*2 operon is composed of six genes that encode two other ArsR, two ArsC (belonging to different families of arsenate reductases), one ACR3 and one ArsH-like protein. The involvement of *ars *operons in arsenic resistance was confirmed by cloning both of them in an *Escherichia coli ars*-mutant. The *ars*1 operon conferred resistance to arsenite and antimonite on *E. coli *cells, whereas the *ars*2 operon was also responsible for resistance to arsenite and arsenate. Although *arsH *was not required for arsenate resistance, this gene seems to be important to confer high levels of arsenite resistance. None of *ars*1 genes were detected in the other type strains of genus *Ochrobactrum*, but sequences homologous with *ars*2 operon were identified in some strains.

**Conclusion:**

A new strategy for bacterial arsenic resistance is described in this work. Two operons involved in arsenic resistance, one giving resistance to arsenite and antimonite and the other giving resistance to arsenate were found in the same bacterial strain.

## Background

Arsenicals generated from natural and man-made sources are widely distributed contaminants of freshwater, groundwater and seawater. The biological availability and physiological and toxicological effects of arsenic depend on its chemical form and oxidation state; the organic forms are the less toxic. Among inorganic forms, arsenite [As(III)] is reported to be on average 100 times more toxic than the less mobile arsenate [As(V)] for most biological systems [1]. However, the toxicity of arsenic to organisms also depends on both endogenous factors (e.g. the membrane oxyanion uptake and efflux pumps) and exogenous factors (e.g. mobility of arsenic forms) [2].

There are many environments containing high amounts of arsenic. However, many organisms can survive in such sites, including bacteria, fungi, algae and plants. These organisms have developed mechanisms for arsenic resistance. These mechanisms can include arsenite oxidation (*aox *genes), respiratory arsenate reduction (*arr *gene) or arsenic resistance by arsenite extrusion (*ars *gene) for cytoplasm defence. Arsenic resistance (*ars*) genes are found in several microorganisms gram-positive and gram-negative and are located either in the chromosome or in the plasmid. Most *ars *operons consist of three genes: *arsR*, *arsB *and *arsC*, and most of the knowledge about these genes comes from studies of *ars *operons in *Escherichia coli *[3], *Pseudomonas aeruginosa *[4] and *Staphylococcus *species [5,6]. Some *ars *operons such as those carried by *E. coli *plasmids R773 [7] and R46 [8] and *Acidiphilium multivorum *plasmid pKW301 [9] have five genes *arsRDABC*. The *arsR *gene encodes a regulatory protein [10] that control the levels of *ars *operon expression, while *arsD *gene encodes for a protein first identified as a regulator [11] and recently reported as a metallochaperone for the arsenic that enhances resistance by delivering this metalloid to the ArsAB efflux pump [12,13]. The ArsA protein is an arsenite-stimulated ATPase, which forms a complex with ArsB, the transmembrane arsenite efflux pump [14]. ArsC is an arsenate reductase which reduces arsenate to arsenite that can then be pumped out of the cell [15,16]. The second family of arsenite carriers has been much less characterized and includes the *arsB *gene of *Bacillus subtilis *[17] and the *Acr*3 gene from *Saccharomyces cerevisiae *[18]. Another gene, *arsH*, without a clear function described, has been found close to arsenic resistance genes in *Yersinia enterocolitica *[19], in *Acidothiobacillus ferroxidans *[20], in *Serratia marcescens *[21], in *Synechocystis *sp. [22], *Sinorhizobium meliloti *[23,24] and *Shigella flexneri *[25]. In addition to the above mentioned arsenic resistance operons, a broad diversity of *ars *operons have been described in different species [26-31].

Species belonging to the genus *Ochrobactrum *have been isolated from clinical and from environmental samples. *Ochrobactrum tritici *SCII24 was isolated from soil by Lebuhn *et al*. [32] however, either its oxyanion resistance or its genetic characterization were not reported. In this paper, we studied the ability of this strain to resist arsenic and antinomy species, and we report the identification of the genes involved in arsenic resistance in *O. tritici*. We also describe the expression of the *ars *genes in *E. coli *AW3110 and discuss the function of each *ars *gene.

## Results

### Arsenic resistance of *Ochrobactrum tritici *SCII24^T^

Tolerance to arsenite, arsenate and antimonite were tested in *O. tritici *SCII24. This strain was able to grow in presence of As(III) up to 50 mM or up to 10 mM Sb(III), and also demonstrated high arsenate resistance, growing at concentrations higher than 200 mM (solubility level of arsenate in this medium).

### Nucleotide sequence of the *ars *genes

**Figure 1:**
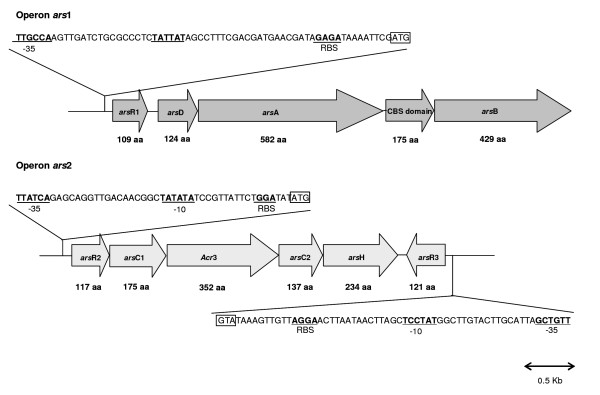
**Genetic organization of the two arsenic resistance clusters in strain *O. tritici *SCII24**. Gene orientations are shown by arrows. Within the predicted structure of the promoters, the -35, -10 regions and ribosome binding sites (RBS) are boldfaced and ATG codons are in boxes.

**Figure 2:**
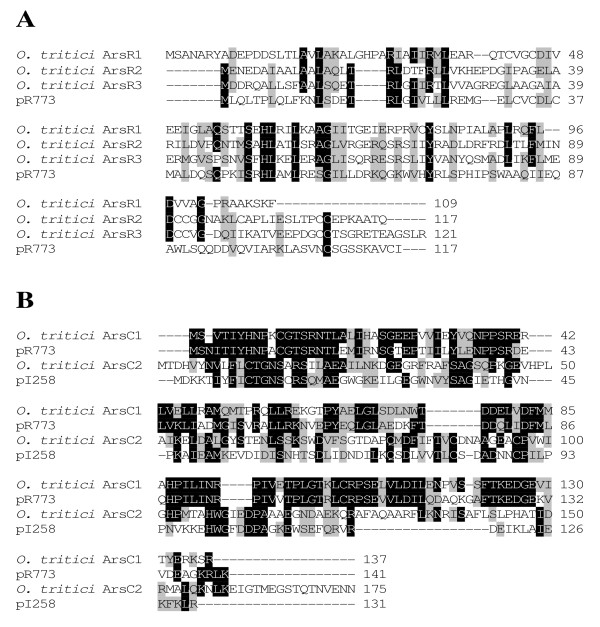
**Alignment of ArsR (A) and ArsC (B) proteins**. The three ArsR sequences from *O. tritici *were aligned with ArsR of *E. coli *pR773 (P15905). Both ArsCs from *O. tritici *were aligned with ArsC homologues from *E. coli *pR773 (AAA21096) and *Staphylococcus aureus *pI258 (AAA25638). The multiple alignment was calculated with CLUSTAL W.

A fragment carrying the *arsB *gene and another fragment containing the *arsC *gene were cloned from a gene library, and the sequences of these two gene clusters were determined using specific oligonucleotide primers. Five potential open reading frames (ORFs) were identified in operon *ars*1 and six ORFs were found in operon *ars*2 (Fig. 1). The characteristics of the predicted products of these ORFs are shown in Table 1. Based on the BLAST analysis, the first cluster is composed by four ORFs, which encode proteins that belong to families of well-known proteins involved in arsenic resistance, ArsR, ArsD, ArsA, ArsB and an additional gene coding for a CBS domain (Cystathionine-β-Synthase), usually not related with arsenic resistance. ORF1 (*arsR1*) encodes an arsenite regulatory protein (ArsR) with 109 aminoacids and shows clear homologies to ArsR proteins from different organisms. The ArsR1 from *O. tritici *has two cysteines (Cys42 and Cys45) instead of the typical highly conserved putative helix-turn-helix DNA binding motif (ELCVCDLC) well identified in plasmid R773 of *E. coli *(Fig. 2A). Shi *et al*. [33] indicated that arsenite only interact with two cysteines of this motif, therefore the presence of 2 cysteines should be enough for arsenic interaction and conformational change of the protein. The second gene, *arsD *encodes a protein of 124 amino acids, which is homologous of the ArsD proteins recently reported as metallochaperone [11,12]. Like others ArsD, this protein also has two pairs of vicinal cysteines, Cys12–Cys13 and Cys112–Cys113, normally involved in inducer binding. The next gene of the group, *arsA *comprises 582 aminoacids, and was described as encoding an *ars *anion-translocating ATPase (ArsA). ArsA consists of two homologous halves, N-and C-terminal domains connected by a short linker sequence. Each domain contains a consensus sequence (GKGGVGKT) for ATP binding and a metalloid binding domain (Cys113, Cys172, Cys422, His148, His451 and Ser420), in agreement with what it was observed for ArsA proteins of other organisms. The next ORF encodes a 175 aa protein with identity to CBS-binding domains of several families of proteins. The last gene of this cluster (*arsB*) encodes a putative arsenite efflux transporter ArsB, with 430 aminoacids.

**Table 1:** Size and putative function of *ars *genes found in strain *O. tritici *SCII24^T^.

**ORF**	**N°. of amino acids**	**Predicted size (KDa)**	**Closest relationship to a known protein (accession n°.)**	**Putative function**
*arsR1*	109	11.7	58% identity and 77% similarity to ArsR from *Anaeromyxobacter sp*. Fw109-5 (ABS27824)	Regulatory protein
*arsD*	124	13.3	68% identity and 83% similarity to ArsD from *Alcaligenes faecalis *(AAS45116)	Regulatory/chaperone protein
*arsA*	582	62.6	98% identity and 99% similarity to ArsA from *Paracoccus methylutens *(AAS87613)	Oxyanion-translocating ATPase
*cbs *domain	151	16.6	99% identity and 99% similarity to CBS domain-like protein from *Paracoccus methylutens *(AAS87612)	Unknown
*arsB*	430	45.3	84% identity and 90% similarity to ArsB from *Alcaligenes faecalis *(AAS45119)	Arsenite membrane pump
*arsR2*	117	12.8	75% identity and 87% similarity to ArsR from *Agrobacterium tumefaciens *str.C58 (AAK87283)	Regulatory protein
*arsC1*	175	19.0	72% identity and 87% similarity ArsC from *Rhizobium leguminosarum bv. Viciae 3841 *(CAK08374)	Arsenate reductase
*Acr3*	352	37.9	82% identity and 89% similarity to ACR3 from *Nitrobacter winogradskyi *Nb-255 (ABA06373)	Arsenite efflux pump
*arsC2*	137	15.6	74% identity and 83% similarity to ArsC from *Mesorhizobium *sp. BNC1 (BAG61184)	Arsenate reductase
*arsH*	241	27.3	75% identity and 82% similarity to NADPH-dependent FMN reductase from *Rhodobacter sphaeroides *ATC17025 (ABP70300)	Unknown
*arsR3*	121	13.2	72% identity and 87% similarity to *Sinorhizobium meliloti *1021 (CAC49416)	Regulatory protein

The identity and similarity values are based on amino acid sequence data.

The nucleotide sequence of the second cluster of the *ars *genes revealed six ORFs that corresponded to *arsR2*, *arsC1*, *Acr3*, *arsC2*, *arsH *and *arsR3*. The first gene of this group encoded a transcriptional regulator belonging to ArsR family without the typical CXCXXC arsenite-binding motif of pR773 ArsR (Fig. 2A). The next gene, *arsC1*, encoded a predicted protein of 137 aa, which is homologous of the ArsC protein family represented by *E. coli *pR773 (Fig. 2B), that uses glutathione and glutaredoxin as electron sources. The third gene was a putative arsenite carrier homologous to ACR3 protein and comprises 352 aa. The ORF4 of this cluster was a second *arsC *gene, encoding a protein (175 aa) with sequence similarity to the thioredoxin-dependent arsenate reductases related to the low molecular weight phosphatase family. In fact, this type of *arsC *comes from evolution of ancient tyrosine phosphatase protein. Downstream of the *arsC2 *gene was found a homologous of a gene previously identified as *arsH*. This gene was recently identified in a few *ars *operons, although its function remains unclear. Recently, published work demonstrates that ArsH protein is an atypical flavodoxin with a non-canonical FMN binding site that catalyzes oxidation of NADPH, generating H_2_O_2 _and with a low azoreductase activity [24,25]. The last ORF also showed similarity with a family of arsenic regulatory proteins and therefore it was named *arsR3*. Unlike all the other genes this protein is divergently transcribed. Alignments of the three different ArsR proteins (ArsR1, ArsR2 and ArsR3) revealed that ArsR2 was more similar to ArsR3 (36% identity and 55% similarity) than to ArsR1 (33% identity and 48% similarity). However, no significant similarity was found between ArsR1 and ArsR3 or between ArsC1 and ArsC2 proteins. "Neural Network Promoter Prediction" [34] was used to predict the location of the putative *ars *promoters. In *ars*1 operon one motif, predicted as a promoter, was identified upstream of *arsR1 *gene. As shown in Fig. 1, the putative -10 region (TATTAT) was separated from the -35 region (TTGCCA) by 17 nucleotides. In *ars*2 operon, two motives estimated as putative promoters were found upstream and downstream of *arsR2 *and *arsR3 *genes, respectively. In this case, we also identified the putatives -10 (TATATA and TATCCT) and -35 (TTATCA and TTGTCG) promoter boxes separated by 17 nucleotides.

### Expression of *O. tritici ars *gene products in *E. coli*

**Figure 3:**
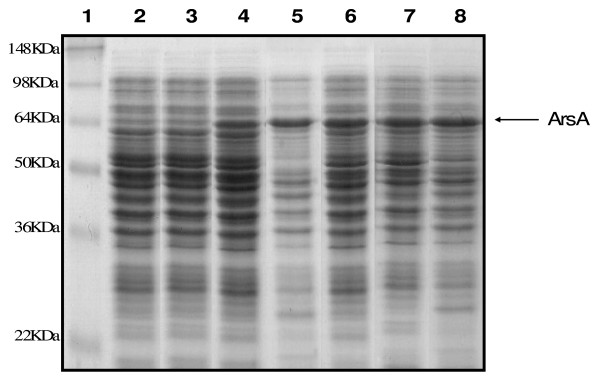
**Expression of the *O. tritici ars *genes in *E. coli *AW3110 under control of *ptrc *promoter**. The gel used was a SDS-12% polyacrylamide gel. Lane1, marker proteins, lane2, plasmid pTRC 99A without an insert; lanes 3, 4 and 5, construct p*arsRDAcbsB *in absence of any oxyanion, in presence of 1 mM As(III) and 1 mM Sb(III), respectively; lanes 6, 7 and 8, construct p*arsDAcbsB *without any oxyanion, in presence of 1 mM As(III) and 1 mM Sb(III), respectively.

Before investigating the role of *O. tritici ars *genes in arsenic resistance, we examined which polypeptides were expressed in *E. coli *by using a polyacrylamide gel. When the transformant carrying the complete *ars*1 operon was cultivated in the presence of arsenite or antimonite, only one additional polypeptide was produced in large amount (Fig. 3). This protein identified by comparison as ArsA, had approximately 63 KDa that was consistent with the size predicted from the aminoacid sequence (62.6 KDa).

However, this protein was not found when the same clone was grown in the absence of arsenite or antimonite. Furthermore, all different clones without *arsR1 *gene cultivated in the presence or absence of these chemicals were always able to produce this ArsA protein, indicating that ArsR worked as a repressor protein in *E. coli*. In contrast, all of the others *ars *products (ArsR1, ArsD, CBS domain, ArsB, ArsR2, ArsC1, ACR3, ArsC2, ArsH and ArsR3) were not found in the total proteins of *E. coli*.

### Ability of the cloned *O. tritici ars *gene products to confer arsenic and antimony resistance in *E. coli *AW3110

**Figure 4:**
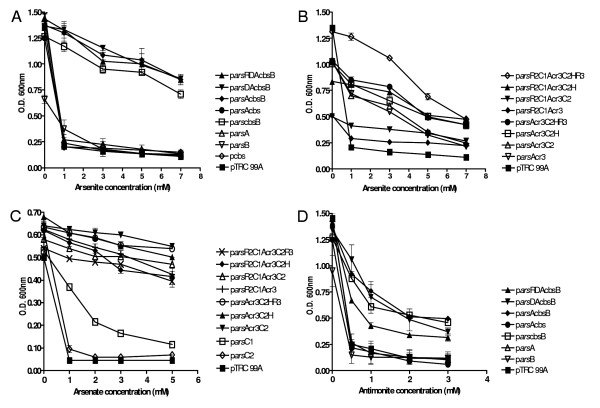
**Growth of *E. coli *AW3110 containing different constructs in the presence of arsenite (A and B), arsenate (C) and antimonite (D)**. Arsenite and antimonite resistance assays were performed in LB medium and arsenate growths were performed in low-phosphate medium. Each data point represents the results of three independent assays. The error bars indicate standard deviations. O.D. 600 nm, optical density at 600 nm.

Different constructs of the *ars *genes were used to evaluate the role of these genes in arsenic resistance in *E. coli *AW3110. All experiments were done in the presence of 0.5 mM IPTG as inducer and the cultures were analyzed for their ability to grow on increasing concentrations of arsenite, arsenate and antimonite (Fig. 4). Cells carrying the constructs from *ars*1 operon p*arsRDAcbsB*, p*arsDAcbsB*, p*arsAcbsB *and p*arscbsB *showed equivalent levels of resistance to As(III) (Fig. 4A). On the other hand, resistance to arsenite was not found in cells carrying the empty vector, or containing the constructs with each of the following genes: *arsA *(p*arsA*), *arsB *(p*arsB*), *cbs *(p*cbs*), *arsA *and *cbs *(p*arsAcbs*).

The experiments related to arsenite resistance by *ars*2 operon, showed that this second operon also conferred As(III) resistance to *E. coli *cells, but at a lower level than the resistance conferred by operon *ars*1. Comparing the different constructs of *ars*2 operon (Fig. 4B), the clone with all genes (p*arsR2C1Acr3C2HR3*) showed the highest As(III) resistance. Cells carrying the constructs p*arsR2C1Acr3C2H*, p*arsAcr3C2HR3 *and p*arsAcr3C2H *showed equivalent levels of resistance to As(III). The constructs p*arsAcr3C2 *and p*arsAcr3 *conferred similar resistance, although at a lower level than the previous constructs and this was more evident for high As(III) concentrations. When the experiments were conducted in the presence of arsenate, we observed that only the cloned *ars2 *genes increased the level of resistance of the host strain. Therefore, abilities of p*arsR2C1Acr3C2HR3 *and subclones to confer resistance to arsenate were tested (Fig. 4C). All the combinations of the multiple *ars *genes tested conferred similar levels of resistance to As(V). Constructs containing the individual genes did not confer resistance to the host strain except for cells that expressed the *arsC *gene, which showed a slight resistance to this oxyanion. These experiments are in agreement with the notion that *arsC*s encode for arsenate reductases, involved in As(V) detoxification. The *ars*1 operon was also responsible for antimony resistance, but the *ars*2 operon was not able to confer resistance to this oxyanion. The figure 4D shows that clones, p*arsDAcbsB*, p*arsAcbsB *and p*arscbsB *conferred Sb(III) resistance to *E. coli *cells, as well as the construct p*arsRDAcbsB *although at an intermediary level. Clone carrying only *arsB *grew less than the wild type in the absence of metal and was not resistant to arsenite. One possibility is the toxicity of the ArsB protein in *E. coli *[9] since in the absence of IPTG, clone carrying only p*arsB *grew as well as the will type (data not shown). On the other hand, p*arscbsB *did not induce toxicity in *E. coli *and clone was arsenite resistant. It is possible that the co-transcription of CBS is required for a correct function of ArsB. The *cbs *gene encodes a protein with a CBS domain that probably dimerizes to form a stable globular structure with ArsB [35].

### Gene induction experiments

**Figure 5:**
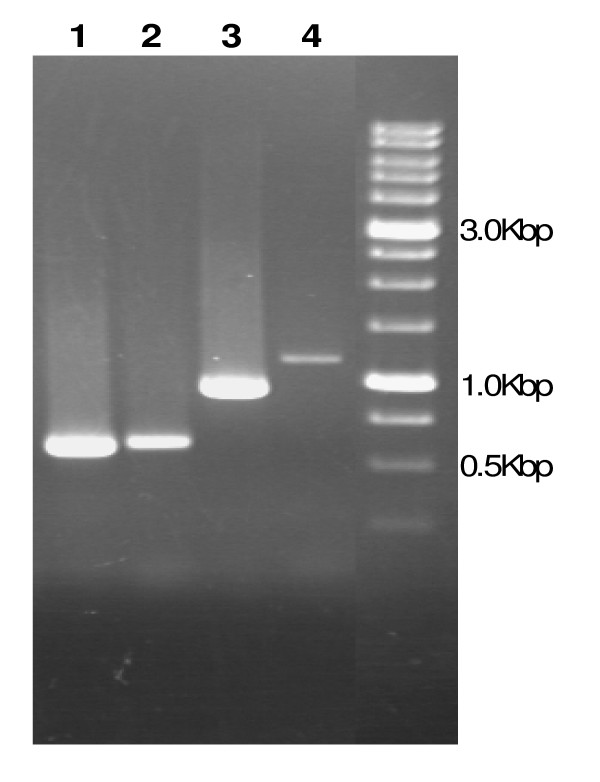
**RT-PCR analysis of *ars*1 genes of *O. tritici *SCII24**. Total RNA isolated from *O. tritici *cells in the exponential phase was used as template in a reverse transcriptase reaction using the reverse primer from *arsB *to generate cDNA. Then, the several intergenic regions were amplified: *arsR*-*arsD *(ane1), *arsD*-*arsA *(lane2), *arsD*-*cbs *(lane3) and *cbs*-*arsB *(lane 4).

RT-PCR was used to test if each *ars *gene cluster forms a unique transcriptional unit. RT-PCR products related to all *ars*1 genes were obtained showing that genes were transcribed from one independent mRNA (Fig. 5). The RT-PCR experiments for *ars*2 also showed products for all genes demonstrating that all genes were transcribed from the same operon (data not shown).

### Detection of *ars *genes in other *Ochrobactrum *strains by Southern blot

**Figure 6:**
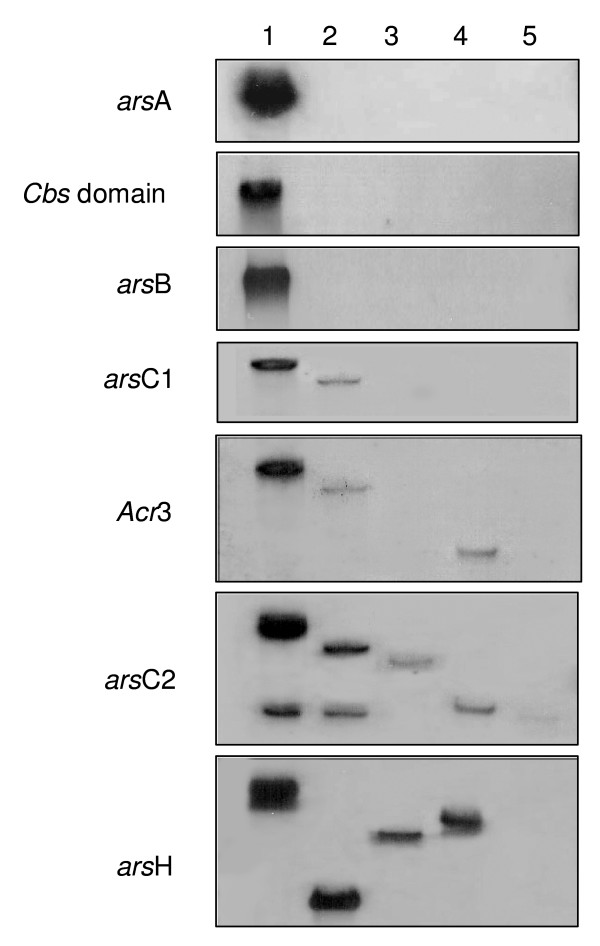
**Southern blot analysis of *ars *genes in *Ochobactrum *strains**. Panels contain hybridization results for *arsA*, gene coding for CBS domain, *arsB*, *arsC1*, *Acr3*, *arsC2 *and *arsH *genes. Lanes:1, *O. tritici *SCII24; 2, *O. tritici *5bvl1; 3, *O. grignonense *OgA9a; 4, *O. anthropi *LMG 3331; 5. *O. intermedium *LMG 3301

Specific probes for structural *ars *genes (*arsA*, *cbs *domain, *arsB*, *arsC1*, *Acr3*, *arsC2 *and *arsH*) were designed to determine the presence of the *ars *genes in other strains belonging to the genus *Ochrobactrum*. Total DNA from these strains was analyzed by Southern blot (Fig. 6). The genes *arsA*, *arsB *and the gene coding for the CBS domain were only detected in strain *O. tritici *SCII24 and the *arsC1 *gene was detected in both strains of *O. tritici *(SCII24 and 5bvl1). Using the *Acr3 *probe a hybridization signal was obtained from preparations of *O. tritici *(SCII24 and 5bvl1) and type strain *O. anthropi*. Southern blot experiments performed with *arsC2 *and *arsH *probes yielded a signal for all of the *Ochrobactrum *strains tested except for type strain *O. intermedium*.

## Discussion

The ability of strain *O. tritici *to resist up to 50 mM As(III), 10 mM Sb(III) and more than 200 mM As(V), turns this bacterium into one of the most resistant microorganisms described to date, most probably due to the presence of two functional *ars *operons (*ars*1 and *ars*2) even if the presence of other mechanisms in the cell, participating in arsenic resistance, can not be overruled. The *ars*1 operon conferred resistance to arsenite and antimonite and *ars*2 was responsible for resistance to arsenite and arsenate. Therefore, *O. tritici *is the first bacteria characterized which contains 2 operons involved in arsenic resistance each one conferring resistance to different metals or metal states. Although, several arrangements of arsenical resistance operons are possible in Bacteria, usually the genes that confer resistance to As(III) and As(V) are present in the same operon [reviewed in [15,16,36]]. In *O. tritici *the *ars *operons showed an unusual genetic composition. The organization of *ars*1 operon displayed some similarities with the well-known *ars *operon on the plasmid pR773 [7] and pR46 of *E. coli *[8], including the genes *arsR*, *arsD*, *arsA *and *arsB*. However, the *arsC *gene (arsenate reductase) was not identified in the *O. tritici ars*1 operon. A second atypical feature was the presence of one additional ORF usually not associated with *ars *genes, which codes a protein with a CBS domain. CBS domains are small domains of unknown function, which were only reported on *ars *genes cluster of *Acidithiobacillus caldus *present on a Tn21-like transposon [26] and on transposon, TnLfArs of *Leptospirillum ferriphilum *[27].

The *ars*2 operon of *O. tritici *could be also differentiated from previously described *ars *operons. One of the differences was the presence of two *arsR *genes with opposite orientations. While *arsR2 *gene was located upstream with the same orientation as *arsC1Acr3C2H*, the *arsR3 *was located downstream and was divergently transcribed. Another intriguing characteristic was the presence of two arsenate reductase genes, *arsC1 *and *arsC2*, encoding distinct ArsC protein families. The ArsC1 belong to the family represented by the *E. coli *pR773 ArsC that uses glutathione and glutaredoxin as electron sources [37]. In contrast, the ArsC2 belong to the family represented by the *S. aureus *pI258 ArsC that uses thioredoxin as an electron source [38]. The *arsR *is commonly found in *ars *operons and its regulatory function, towards the basal operon expression, has been demonstrated [5,9,30]. For most of these trans-acting ArsR regulatory proteins, even with proteins with low homology, the consensus sequence for metal binding (ELC32VC34DLC37) was found. However, the three ArsRs from *O. tritici *had a very low or even no homology between them and they did not show the typical arsenite-binding motif (Fig. 2). The absence of conserved metal binding box was also previously identified in *Corynebacterium glutamicum *[29] and in *Acidithiobacillus ferroxidans *[20]. In the case of *A. ferroxidans*, Qin *et al*. [39] proved the involvement of a vicinal cysteine pair, Cys95, Cys96 and a third residue Cys102, in the transcriptional regulation by trivalent metalloids. In *O. tritici*, it was apparent that ArsR1 could function as a repressor since the expression of the *arsA *gene (visible from SDS polyacrylamide gel) depended on the presence of the inducer. The most probable regulatory role of the remaining *arsR*s genes could not be evaluated, because the *ars*2 operon products were not detected by polyacrylamide gel. The *arsD*, together with the *arsR*, has been associated to the control of the maximal level of *ars *operon expression preventing the overexpression of ArsB [40], which is toxic in excess. Our results also illustrate the toxic effect of ArsB, since the overproduction of this arsenite efflux pump (ArsB) had a deleterious effect on *E. coli *cells. The experimental data of this work do not support the regulatory function of the ArsD over ArsB expression, since *E. coli *cells that did not carry the *arsD *gene (constructs p*arsAcbsB *and p*arscbsB*) showed similar resistance as the constructs with *arsD*, although the role of metallochaperone-mediator can not be excluded. The *arsB *gene product could not be detected in *E. coli *by SDS-PAGE as was previously reported by other authors [9]. Nevertheless, in contrast to other operons [4,41], the *O. tritici arsB *gene alone was not able to confer resistance to arsenite and antimony. However, the presence of the CBS domain in addition to *arsB *gene was enough to increase the resistance to these metalloids. CBS domains are found in a wide range of other unrelated proteins and in some of them a regulatory role of the protein structure has been suggested [35]. Therefore, in *O. tritici *it is possible that the protein with identity to CBS-binding domain may play a role as a structure decreasing the toxicity of the ArsB. As we mentioned before the *ars*2 operon also conferred resistance to As(III), although the *ars*2 operon was the only one responsible for arsenate detoxification. A second arsenite efflux pump was located in the *ars*2 operon, but, differently from *arsB*, this one was phylogenetically near to arsenite transporter familly-ACR3, represented by *Saccharomyces cerevisiae *[18]. *O. tritici *ACR3 protein as well as yeast Acr3p catalyzes extrusion of arsenite from the cells conferring arsenite but not antimonite resistance. As expected, ACR3 was not homologous to the ArsB found in operon *ars*1, even though they both function as arsenite efflux pumps.

Arsenate resistance in *O. tritici *could involve only one or both *arsC *genes to convert As(V) to As(III), since we observed that not only the ArsC1 was functional in *E. coli*, but also the ArsC2 usually related to gram-positive ArsC family. ArsC reductases from different phylogenetic groups were already showed to be functional in *E. coli *[41]. The arsenite produced was then pumped out from the cells by ACR3 protein. An *arsH*-like gene was also located in operon *ars*2. The gene *arsH *was first described as a regulator [19] but no specific role could be conferred to *arsH *in *A. ferroxidans *[20] and in plasmid R478 of *S. marcescens *[21]. Recently, in *Sinorhizobium meliloti *[24] and in *Shigella flexneri *[25], ArsH was described as a H_2_O_2_-forming NADPH:FMN oxidoreductase that also reduces azo dyes. *O. tritici arsH *gene did not have any effect in arsenate resistance and was not fundamental to arsenite resistance, however, the removal of this gene resulted in a reduction of arsenite resistance by *E. coli *cells in the presence of high levels of As(III). Therefore, a similar role to that proposed for the *Sinorhizobium *ArsH can be expected since homology studies of ArsH from *O. tritici *showed that the protein belongs to the same NADPH-dependent FMN reductase family.

Southern blotting did not reveal *ars*1-homologous sequences in the other type strains of the genus *Ochrobactrum*, but signed the presence of the *ars*2 genes in some of these bacteria. It is interesting to note the concurrent absence of the first cluster of genes along with strain-sensitivity to As(III) and Sb(III), suggesting the involvement of *ars*1 operon in detoxification of both oxyanions in these strains. In contrast, the detection in *Ochrobactrum *strains of genes from *ars*2 operon along with arsenate resistance may reflect the relation between the presence of *ars*2 genes and arsenate resistance.

## Conclusion

This work illustrates the presence of operons *ars*1 and *ars*2 in *O. tritici *and its possible involvement in arsenic resistance showed by *E. coli *heterologous complementation analysis. Operon *ars*1 seems to code for the main arsenite detoxification system and is entirely responsible for antimonite resistance in this strain. Operon *ars*2 encodes a group of different proteins with the arsenate detoxification as main function, and additionally is involved in arsenite resistance. This operon includes genes coding for arsenate reductases with different phylogenetic origins.

## Methods

### Strains, plasmids and culture conditions

The strains and plasmids used in this study are listed in Table 2. *E. coli *XL1-Blue and *E. coli *AW3110 were used as hosts for the cloning vectors or expression vectors. *E. coli *was grown in LB medium containing, per litre, 10 g of tryptone, 5 g of yeast extract, and 5 g of NaCl. Ampicillin (100 μg/ml) and isopropyl-thio-ß-D-galactoside (IPTG) (0.5 mM) was added to the medium, as required. For some experiments, cells were grown in a low-phosphate medium.

**Table 2:** Bacterial strains and plasmids used.

**Strain or plasmid**	**Relevant characteristic(s)**	**Reference or source**
Strains		
*O. tritici *SCII24^T^	As(III)^r^, As(V)^r^, Sb(III)^r^	LMG
*O. tritici *5bvl1	As(III)^s^, As(V)^r^, Sb(III)^s^	This laboratory
*O. grignonense *OgA9a^T^	As(III)^s^, As(V)^r^, Sb(III)^s^	LMG
*O. anthropi *LMG 3331^T^	As(III)^s^, As(V)^r^, Sb(III)^s^	LMG
*O. intermedium *LMG 3301^T^	As(III)^s^, As(V)^r^, Sb(III)^s^	LMG
*E. coli *XL1-Blue	*rec*A1 *end*A1 *gyr*A96 *thi hsd*R17 (r^-^_k_m^+^_k_) *sup*E44 *rel*A1 *lac *[F' *pro*AB^+ ^*lac*I^q^ZΔM15::*Tn*10(tet^r^)]	Stratagene
*E. coli *AW3110	K-12 F- IN(rrnD-rrnE)Δ*ars*::cam	3
Plasmids		
pGEM-T Easy	Amp^r ^lacZ, cloning vector	Promega
pUC18	Amp^r ^lacZ, cloning vector	Invitrogen
pTRC 99A	Amp^r^, *ptrc *promotor, expression vector	Amersham
p*arsR1DAcbsB*	Fragment of genes *arsR1, arsD*, *arsA*, *arsB *and *cbs *domain cloned into pTRC 99A	This study
p*arsDAcbsB*	Fragment of genes *arsD*, *arsA *and *arsB *and *cbs *domain cloned into pTRC 99A	This study
p*arsAcbsB*	Fragment of genes *arsA*, *arsB *and *cbs *domain cloned into pTRC 99A	This study
p*arsAcbs*	Fragment of genes *arsA *and *cbs *domain cloned into pTRC 99A	This study
p*arscbsB*	Fragment of genes *arsB *and *cbs *domain cloned into pTRC 99A	This study
p*arsA*	Fragment of gene *arsA *cloned into pTRC 99A	This study
p*arsB*	Fragment of gene *arsB *cloned into pTRC 99A	This study
p*arsR2C1Acr3C2HR3*	Fragment of genes *arsR2*, *arsC1*, *Acr3*, *arsC2*, *arsH *and *arsR3 *cloned into pTRC 99A	This study
p*arsR2C1Acr3C2H*	Fragment of genes *arsR2*, *arsC1*, *Acr3*, *arsC2 *and *arsH *cloned into pTRC 99A	This study
p*arsR2C1Acr3C2*	Fragment of genes *arsR2*, *arsC1*, *Acr3 *and *arsC2 *cloned into pTRC 99A	This study
p*arsR2C1Acr3*	Fragment of genes *arsR2*, *arsC1 *and *Acr3 *cloned into pTRC 99A	This study
p*arsAcr3C2HR3*	Fragment of genes *Acr3*, *arsC2*, *arsH *and *ars*R2 cloned into pTRC 99A	This study
p*arsAcr3C2H*	Fragment of genes *Acr3*, *arsC2 *and *ars*H cloned into pTRC 99A	This study
p*arsAcr3C2*	Fragment of genes *Acr3 *and *arsC2 *cloned into pTRC 99A	This study
p*arsC1*	Fragment of gene *arsC1 *cloned into pTRC 99A	This study
p*arsC2*	Fragment of gene *arsC2 *cloned into pTRC 99A	This study

LMG, Laboratorium voor Microbiologie, Universiteit Gent

This medium contains in g/l, Tris 6.06 (pH 7.0), NaCl 4.68, KCl 1.49, NH_4_Cl 1.07, Na_2_SO_4 _0.43, MgCl_2_.6H_2_O 0.2, CaCl_2_.2H_2_O 0.03, Na_2_HPO_4_.12H_2_O 0.23 and glucose 5.0. Analytical-grade salts of NaAsO_2_, KH_2_AsO_4 _and C_4_H_4_KO_7_Sb.0.5H2O (Sigma) were used to prepare 0.5 M stock solutions. Metalloid-containing medium was solidified with 20 g of Bacto-Agar (Difco Laboratories) per litre.

### Construction of an *O. tritici *SCII24T gene library

The following degenerate pairs of 5 oligonucleotides were designed based on conserved regions of several prokaryotes arsenic resistance genes, *arsB *and *arsC*, available from public databases: forward primers 5'-GTG/CATC/TTGGCAA/GCCG/CAAA/GGG-3' and 5'ACG/CATC/TTAC/TCAC/TAAC/TCCG-3'; reverse primers 5'GTG/CGGCATA/GTTA/GTTCATA/GAT-3' and 5'TCGCCA/GTCC/TTCC/TTTG/CGTA/GAA-3', respectively. PCR fragments of 0.97 Kb for *arsB *gene and 0.35 Kb for *arsC *gene were amplified from *O. tritici *with these primers (purchased from Sigma Genosys). These fragments were subsequently used as probes for the isolation of the complete gene from the library. Total DNA was isolated from *O. tritici *as previously described [42]. Two partial genomic libraries from this strain, containing DNA fragments selected by Southern blot analysis of genomic DNA with the 0.97 Kb and 0.35 Kb fragment probes, were obtained by complete digestion of total DNA with restriction enzyme *Hind*III (Roche Molecular Biochemicals), followed by ligation of purified fragments of around 5–9 Kb into the *Hind*III site of pUC18 (Invitrogen) and subsequent transformation of *E. coli *XL1-Blue with the ligation mixture. Clones were selected for their resistance and positive clones were confirmed by colony hybridization with a digoxigenin-labeled probe (Roche Molecular Biochemicals). Inserts of positive pUC18 clones and pGEM-T Easy (Promega) clones were sequenced by using vector-and insert-specific oligodeoxynucleotide primers. DNA sequencing was performed by using a BigDye Terminator v1.1 Cycle Sequencing Kit (Applied Biosystems) using a model ABI310 automatic DNA sequencer. Nucleic acid and protein sequence analyses were performed with BioEdit editor [43]. Comparison searches were performed by using the BLAST program of the National Center for Biotechnology Information (NCBI).

### Identification of the *ars *gene products

The *ars *genes were amplified by PCR using specific forward and reverse primers containing additional *Xba*I and *Hind*III recognition sites, respectively. The PCR products were purified after digestion with *Xba*I and *Hind*III and ligated into the corresponding sites into the pTRC 99A expression vector (Amersham Biosciences). Then, each construction was transformed into competent *E. coli *AW3110. Cells bearing each plasmid were grown at 37°C overnight in LB medium containing ampicillin. Overnight cultures were diluted 100-fold into fresh LB medium containing ampicillin alone or with arsenite, or with arsenate, or with antimonite. When the absorbance at 600 nm reached 0.8 to 1.0, 0.5 mM IPTG was added to the culture to induce gene expression, and cultivation was continued for 6 hours. Cells were pelleted by centrifugation and dissolved in a loading buffer for sodium dodecyl sulfate (SDS)-polyacrylamide gel electrophoresis (PAGE). Total proteins were analyzed on 12% – 15% polyacrylamide gels.

### Arsenic and antimony resistance assays

Arsenite and antimonite resistance assays were carried out in LB medium, while for arsenate resistance, cells were grown in low phosphate medium. Overnight cultures were diluted 100-fold into fresh medium containing the ampicillin and different concentrations of arsenite, antimonite and arsenate. The bacterial suspensions were incubated at 37°C with shaking for 5 hours for experiments in LB medium or for 15 hours for assays performed in low phosphate medium and the absorbance at 600 nm was measured.

### RNA isolation and RT-PCR experiments

Total RNA was obtained from mid exponential phase *O. tritici *cells grown in low-phosphate medium exposed for 2 h to 1 mM of arsenite, antimonite or arsenate. Control cells were not exposed to added compounds. Total RNA isolated from those cells by the Rneasy Mini kit (Qiagen), according to the manufacturer's instructions, was then digested with RQ1 Rnase-free DNase (Promega) to remove the residual DNA. cDNA synthesis was done with Sensiscript Reverse Transcriptase (Qiagen) according to the manufacturer's instructions. Briefly, the reverse primers from *arsB *and *arsH *genes were used to create the cDNA for each *ars *gene clusters. cDNAs were used as template for the second PCR amplification reaction. For monitoring the expression of *ars*1, were used 5 μl of the RT reaction and specific primers designed to amplify a region spanning the junction of the different genes of operon *ars*1 and operon *ars*2. RT-PCR products were examined by 1% agarose gel electrophoresis. DNA contamination of the mRNA was determined by PCR using Taq polymerase without reverse transcriptase.

### Southern hybridization

Southern blot analysis was performed as described by Sambrook *et al*. [42]. Purified DNAs (10 μg) of strains *O. tritici *SCII24, *O. tritici *5bvl1, *O. grignonense *OgA9a, *O. anthropi *LMG 3331 and *O. intermedium *LMG 3301 were subjected to digestion with restriction enzymes and electrophoresed on agarose gel [0.8% (w/v)]. DNA was capillary transferred for approximately 16 h to a nylon membrane in 0.4N NaOH, 1 M NaCl buffer followed by neutralization. DNA probes for structural genes [*arsA*, *cbs *domain, *arsB*, *arsC1*, *Acr3*, *arsC2 *and *arsH*] were amplified by PCR using gene-specific primers, recovered from agarose gels and purified with Wizard SV Gel and PCR Clean-Up System (Promega). Each probe was labelled with dioxigenin-dUTP nonradioactive (Roche Molecular Biochemicals) and the subsequent pre-hybridization and hybridization with the membrane were performed at 45°C to 58°C using DIG High Prime DNA Labelling and Detection Starter Kit II, following the manufacturer's instructions. The membrane was washed twice with 2× SSC-0.1% SDS at room temperature and twice with 0.2× SSC-0.1% SDS for 15 min at 68°C by constant agitation. Membranes were autoradiographed after incubation at 37°C for 45 min.

### Nucleotide sequence accession numbers

The nucleotide sequences of the DNA fragments containing the *ars*1 operon and *ars*2 operon have been submitted to GenBank under the accession no. DQ490089 and DQ490090, respectively.

## Authors' contributions

RB performed all experiments, designed the study and participated in the writing of the manuscript. A–PC performed the gene sequencing. PVM coordinated the study, participated in its design and in the writing of the manuscript. All authors read and approved the final manuscript.
